# Efficacy of continuous in-wound infusion of levobupivacaine and ketorolac for post-caesarean section analgesia: a prospective, randomised, double-blind, placebo-controlled trial

**DOI:** 10.1186/s12871-018-0609-2

**Published:** 2018-11-10

**Authors:** Jozica Wagner-Kovacec, Petra Povalej-Brzan, Dusan Mekis

**Affiliations:** 1 Department of Anaesthesiology, Intensive Care and Pain Management, University Medical Centre Maribor, Ljubljanska ulica 5, 2000 Maribor, Slovenia; 2 Faculty for Health Sciences, University of Maribor, Žitna ulica 15, 2000 Maribor, Slovenia; 3 Medical Faculty, University of Maribor, Taborska ulica 8, 2000 Maribor, Slovenia; 4 Faculty of Electrical Engineering and Computer Science, University of Maribor, Koroška cesta 46, 2000 Maribor, Slovenia; 5 Department for Medical Research, University Medical Centre Maribor, Ljubljanska ulica 5, 2000 Maribor, Slovenia

**Keywords:** Caesarean section, Analgesia, In-wound infusion, Levobupivacaine, Ketorolac

## Abstract

**Background:**

In-wound catheters for infusion of local anaesthetic for post-caesarean section analgesia are well tolerated in parturients. Few studies have examined continuous in-wound infusion of a combination of local anaesthetic and non-steroidal anti-inflammatory drug for post-caesarean section analgesia. This single centre study evaluated post-operative analgesic efficacy and piritramide-sparing effects of continuous in-wound infusion of either local anaesthetic or non-steroidal anti-inflammatory agent, or the combination of both, versus saline placebo, when added to systemic analgesia with paracetamol.

**Methods:**

After National Ethical Board approval, 59 pregnant women scheduled for non-emergency caesarean section were included in this prospective, randomised, double-blind, placebo-controlled study. The parturients received spinal anaesthesia with levobupivacaine and fentanyl. Post-operative analgesia to 48 h included paracetamol 1000 mg intravenously every 6 h, with the studied agents as in-wound infusions. Rescue analgesia with piritramide was available as needed, titrated to 2 mg intravenously. Four groups were compared, using a subcutaneous multi-holed catheter connected to an elastomeric pump running at 5 mL/h over 48 h. The different in-wound infusions were: levobupivacaine 0.25% alone; ketorolac tromethamine 0.08% alone; levobupivacaine 0.25% plus ketorolac tromethamine 0.08%; or saline placebo. The primary outcome was total rescue piritramide used at 24 h and 48 h post-operatively, under maintained optimal post-caesarean section analgesia.

**Results:**

Compared to placebo in-wound infusions, ketorolac alone and levobupivacaine plus ketorolac in-wound infusions both significantly reduced post-operative piritramide consumption at 24 h (*p* = 0.003; *p* < 0.001, respectively) and 48 h (*p* = 0.001; *p* < 0.001). Compared to levobupivacaine, levobupivacaine plus ketorolac significantly reduced post-operative piritramide consumption at 24 h (*p* = 0.015) and 48 h (*p* = 0.021). For levobupivacaine versus ketorolac, no significant differences were seen for post-operative piritramide consumption at 24 h and 48 h (*p* = 0.141; *p* = 0.054).

**Conclusion:**

Continuous in-wound infusion with levobupivacaine plus ketorolac provides greater opioid-sparing effects than continuous in-wound infusion with levobupivacaine alone.

**Trial registration:**

German Clinical Trials Register: retrospectively registered on 30 July, 2014, DRKS 00006559.

## Background

Caesarean section is a common surgical procedure that is being performed at increasingly higher rates [1, 2]. Immediate post-operative pain can be higher than for many other types of major surgery [3]. Effective pain relief after caesarean section is very important, as it allows the mother to take care of her new-born baby. Suboptimally treated or persistent post-operative pain can lead to impaired physical function and loss of sleep, and is associated with premature arrest of breastfeeding, inability to care for the new-born, thromboembolism due to delayed ambulation, delayed discharge, parturient dissatisfaction, persistent pain, and postnatal depression [4, 5]. The incidence of persistent pain after caesarean section is 6% to 18% [6], although a recent study showed a low incidence of persistent pain (0.3%) at 12 months after child-birth [7].

The incidence of post-operative pain remains high despite the availability of new drugs and novel analgesic techniques [3], potentially due to the worries about side-effects of the analgesic on the mother and the new-born through analgesic secretion in the breast milk [3]. Inter-individual variations in pain perception also complicate the effectiveness of post-caesarean section analgesia [8, 9]. Pre-operative predictive tests for post-caesarean pain have been studied and might provide the basis for individualised approaches to multimodal post-operative pain management [10, 11]. As adjuncts to neuraxial and systemic analgesics, regional nerve blockade and wound infiltration have been recommended as components of multimodal analgesia for post-caesarean pain relief [12].

The aim of the present study was to evaluate the effectiveness of pain treatment with in-wound infusion of levobupivacaine and/or ketorolac after caesarean section as adjuvant to systemic analgesics. The primary outcome was total rescue piritramide used at 24 h and 48 h post-operatively. Secondary aims were persistent post-operative pain and/or any new skin sensation after 8 weeks, and satisfaction of the parturient with the quality of analgesia.

## Methods

This prospective, randomised, double-blind, placebo-controlled trial was approved by the National Medical Ethics Committee (Republic of Slovenia National Medical Ethics Committee, Number 169/07/11) on 12 July, 2011, and is registered with the German Clinical Trials Registry (DRKS 00006559). Pregnant women scheduled for non-emergency caesarean section were recruited at University Clinical Centre Maribor, Slovenia, from January 2012 to September 2014. All of the participants gave their written informed consent.

The inclusion criteria were: age ≥ 18 years; gestational age ≥ 37 weeks; and American Society of Anesthesiologists (ASA) physical status I or II. The exclusion criteria included: refusal to participate; ASA III or higher; previous caesarean section or abdominal surgery; body mass index > 30 kg/m^2^ before conception; history of drug or alcohol abuse; allergies to local anaesthetics, paracetamol or non-steroidal anti-inflammatory drugs (NSAIDs); and any known contra-indication for neuraxial anaesthesia.

The parturients were seen during the pre-operative visit by the research personnel, evaluated for study inclusion, and approached for recruitment and informed consent. They were instructed regarding the study group allocation, the analgesia system they would use after surgery, the continuous in-wound infusion device, how to use numeric rating scales (NRS), from 0 to 10 for pain (0, none, to 10, worst possible pain), and from 1 to 5 for quality of analgesia. They were also introduced to the persistent post-operative pain questionnaire.

All of the parturients were premedicated with ranitidine 50 mg intravenously (i.v.) and metoclopramide 10 mg i.v., and 30 mL oral 0.3 M sodium citrate. After a 250 mL i.v. preload with 6% hydroxyethyl starch 130/0.4 in 0.9% sodium chloride (saline), the parturients were monitored using standard intra-operative monitoring (i.e., non-invasive blood pressure, electrocardiogram, peripheral oxygen saturation pressure [SpO_2_]). A standardised intrathecal anaesthesia injection was performed for all parturients. With the parturient in the sitting position, a 25-gauge pencil-point spinal needle was inserted in the L_3_-L_4_ interspace, and 2.0 mL to 2.2 mL levobupivacaine 0.5% with fentanyl 15 μg was injected. The parturients were positioned supine, with 15° to 30° left tilt, for prevention of possible aorto-caval compression. A bladder catheter was inserted just before the sensory block reached the T_4–6_ level.

The parturients were randomly allocated to one of the four groups before surgery, according to numbered sealed envelopes. The sealed envelopes were chosen by the parturients and given to the nurse anaesthetists, who prepared an elastomeric pump (On Q Pain Buster; I-Flow Corporation, Lake Forest, CA, USA) in the operating theatre next door. The elastomeric pump was filled with 270 mL of the study solution indicated on each paper in the envelopes, and run at 5 mL/h. The four groups received continuous post-operative infusions of saline with the following additions: levobupivacaine 0.25% alone (LB group); ketorolac tromethamine 0.08% alone (KT group); levobupivacaine 0.25% plus ketorolac tromethamine 0.08% (LB + KT); saline placebo.

Here, levobupivacaine was selected as the local anaesthetic for its low toxicity and analgesic potency, with levobupivacaine 0.25% in continuous in-wound infusion not expected to give any problems of overdosing for the average weight of the parturients. Ketorolac was selected as the NSAID as it is safe and effective for post-caesarean i.v. pain therapy, and safe regarding breastfeeding. The maximal daily i.v. dose of ketorolac should also be safe for subcutaneous infusion.

If the systolic blood pressure of the parturients was < 90 mmHg or if they showed nausea and/or vomiting due to hypotension, phenylephrine 50 μg to 100 μg or ephedrine 5 mg to 10 mg was titrated i.v. until the parturient regained their normal blood pressure. For nausea or vomiting, ondansetron 4 mg was given i.v..

After local subcutaneous infiltration with 20 mL of one of the randomised agents, the skin was closed with a multi-holed 12.5-cm catheter inserted into the wound above the fascia. Continuous infusion of the randomised agent was then started, at 5 mL/h (using an elastomeric pump), and continued for 48 h. The parturient and the staff involved in the peri-operative management and data collection were blinded to the assignment of the parturient to one of the four randomised agents.

The post-operative analgesia protocol to 48 h included paracetamol 1000 mg i.v. every 6 h, along with the studied agents as in-wound infusions. For rescue analgesia, piritramide was available as needed, titrated to 2 mg i.v. until a pain NRS ≤4.

The demographic characteristics and gestational ages were collected for the parturients. Over the first 48 h post-caesarean section, the pain NRS (0–10) was completed (at 3, 6, 12, 18, 24, 36 and 48 h) as specified for the pain at the surgical site at rest and under movement (e.g., raising legs, coughing, walking), for nausea and/or vomiting (yes/no), and for any new skin sensations at the surgical site (yes/no). The time from the end of the operation to the first request for piritramide was recorded. After 72 h, the satisfaction of the parturient for the quality of analgesia for the first 48 h after the caesarean section was determined (NRS 1–5; 1, very bad; 2, bad; 3, good; 4, very good; 5, excellent). After 8 weeks, the parturients were contacted by telephone for completion of a questionnaire about any persistent post-operative pain (Table 1).

**Table 1:** Questionnaire used for persistent post-operative pain

1. Do you feel any pain at the scar area?	
If yes: Do you take medication to alleviate it?	
Do you take analgesics every day or occasionally (at least twice per week)?	
Which one(s)?	
If no: Do you have any particular sensations from the scar area? Itching, burning, sensitivity?	
2. Do you feel pain at any other place?	
If yes: Where?	
Do you take analgesics?	
3. Which unpleasant manifestations have you experienced since your operation?	

The primary outcome was total rescue piritramide used at 24 h and 48 h post-operatively. Secondary aims were persistent post-operative pain and/or any new skin sensation after 8 weeks, and the satisfaction of the parturient with the quality of analgesia.

### Statistical analysis

The required sample size calculation was based on a pilot study of 20 parturients (five for each group). The calculated maximum number of required parturients for each group was 19 to satisfy 80% power of detecting an effect of medium size (0.4) and an α error probability of 0.05. According to this sample size calculation, we planned to enrol 88 participants.

Statistical analysis was performed using SPSS V. 24.0 (IBM). The parturient demographic and parametric data were analysed using ANOVA, with Bonferroni *post-hoc* tests for correction for multiple comparisons. The non-parametric data for the between-group comparisons were analysed using Kruskal Wallis tests and *post-hoc* tests with Bonferroni correction. Chi-square tests were performed to determine the differences among groups for the feeling of persistent pain and for any new skin sensations. Parametric data are presented as means ±standard deviation (SD), and non-parametric data are presented as medians and 95% confidence interval (CI). *p* < 0.05 was considered significant.

## Results

**Fig. 1:**
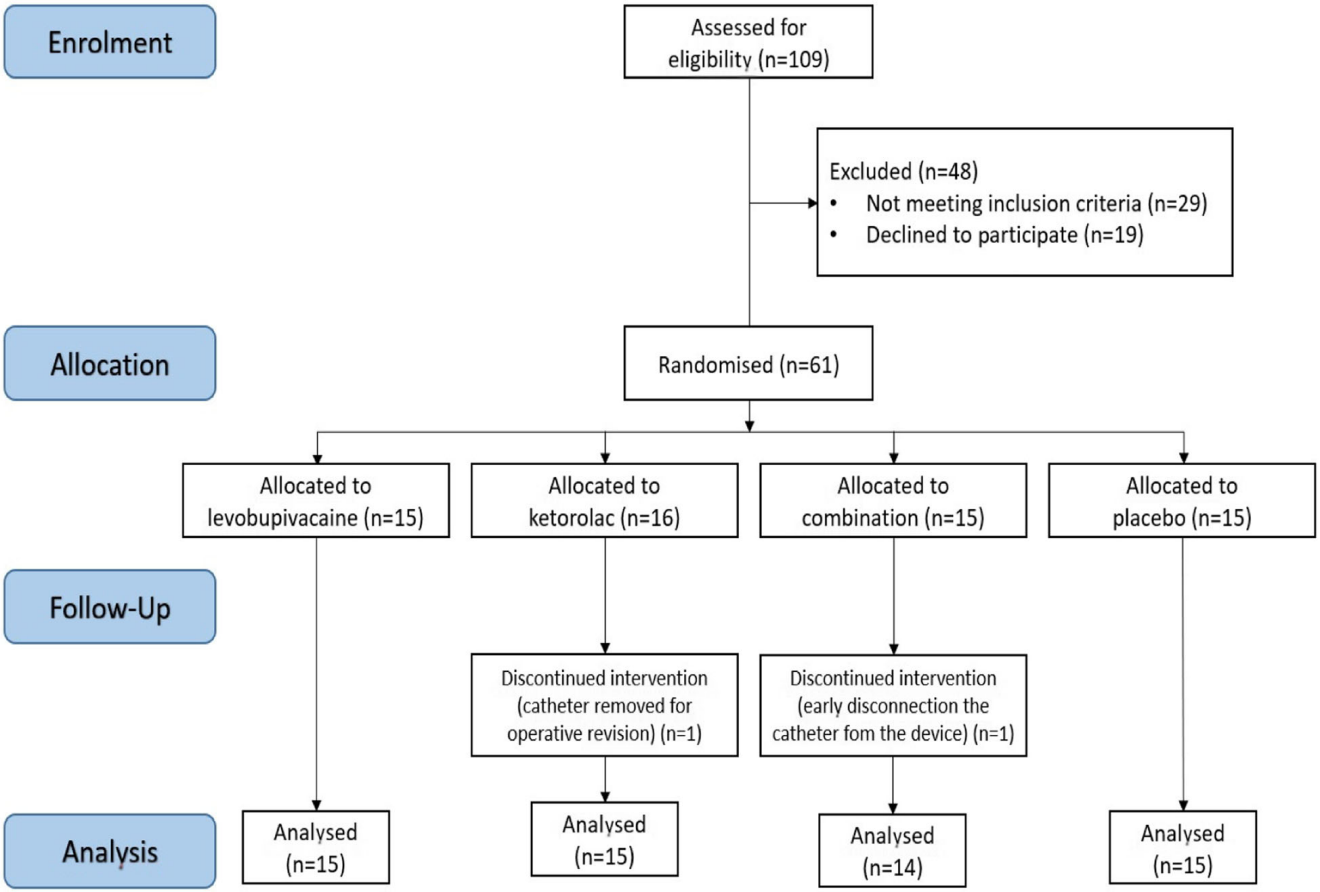
CONSORT flow chart

Fifty-nine participants completed the study and were included in the data analysis. The Consolidated Standards of Reporting Trials (CONSORT) diagram is shown in Fig. 1, and the parturient characteristics are given in Table 2. The groups were compared using Kruskal Wallis tests. Between group comparisons were made using Bonferroni correction. Across the parturient characteristics (i.e., age, weight, height, body mass index, gestational age), there was a significant difference for age (*p* = 0.014) and gestational age (*p* = 0.012) across all of the treatment groups. The between groups analysis showed that the LB group had significantly lower age and gestational age compared to the placebo group. The other groups did not show any significantly differences in these observed characteristics (Table 2).

**Table 2:** Parturient characteristics across the four parturient groups

Characteristic	Levobupivacaine alone (*n* = 15)	Ketorolac tromethamine alone (*n* = 15)	Combination (*n* = 14)	Saline placebo (*n* = 15)	*p*-value
Age (years)	**28.1 ± 2.92** ^a^	31.7 ± 5.11	28.8 ± 5.01	**32.5 ± 3.91** ^a^	0.014^a^ (levobupivacaine vs saline placebo)
Weight at conception (kg)	62.0 ± 10.71	59.4 ± 6.81	64.0 ± 8.56	64.1 ± 5.74	ns
Weight at caesarean section (kg)	74.9 ± 11.50	74.7 ± 9.18	80.2 ± 8.93	79.0 ± 7.60	ns
Height (cm)	167.5 ± 6.06	166.1 ± 4.91	166.5 ± 5.97	165.9 ± 4.22	ns
Body mass index at conception (kg/m^2^)	21.9 ± 3.42	21.2 ± 2.34	22.8 ± 2.46	23.1 ± 2.34	ns
Body mass index at caesarean section (kg/m^2^)	26.7 ± 3.78	27.1 ± 3.09	28.9 ± 2.27	28.7 ± 3.20	ns
Gestational age (weeks)	**38 6/7 ± 0.71** ^a^	38 6/7 ± 0.80	38 5/7 ± 0.67	**39 2/7 ± 0.71** ^a^	0.012^a^ (levobupivacaine vs saline placebo)

Data are means ±SD; ^a^significant difference among groups calculated using ANOVA/Kruskal Wallis test and post-hoc test with Bonferroni correction; ns: non-significant; significant differences are marked with bold text

**Fig. 2:**
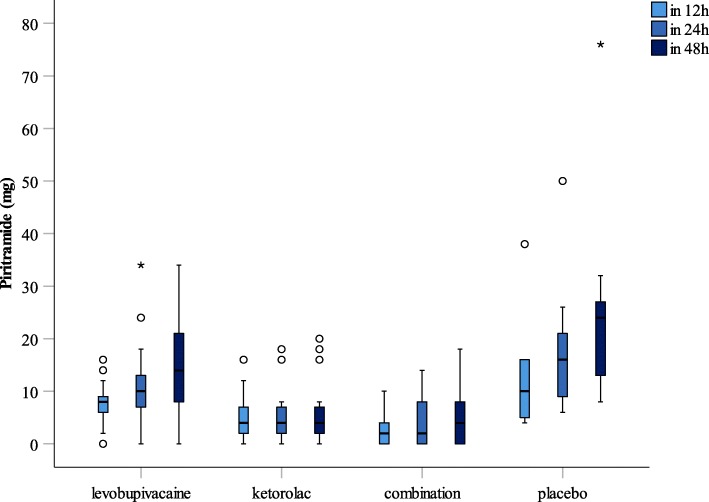
Medians and interquartile ranges across the four parturient groups for the total consumption of piritramide, at 12, 24 and 48 h post-caesarean section. *,° outliers

The total consumption of piritramide for 12, 24 and 48 h across the four groups is given in Table 3, with the median and interquartile ranges for the total consumption of piritramide shown in Fig. 2. Two of the parturients in the KT group and six from the LB + KT group did not request any piritramide in the first 24 h post-caesarean section. One parturient from the KT group and five from the LB + KT group did not request any piritramide to 48 h post-caesarean section.

**Table 3:** Total consumption of piritramide across the four parturient groups, at 12, 24 and 48 h

Parturient group	Time	Total consumption of piritramide (mg)				*p*-value
	(h)	Median	Minimum	Maximum	95% CI	
Levobupivacaine alone	12	8	0	16	8–14	ns
	24	10	0	34	8–14	0.015^a^ (vs combination)
	48	14	0	34	8–22	0.021^a^ (vs combination)
Ketorolac tromethamine alone	12	4	0	16	2–10	ns
	24	4	0	18	4–16	0.003^a^ (vs saline placebo)
	48	4	0	20	4–16	0.001^a^ (vs saline placebo)
Combination	12	2	0	10	2–6	ns
	24	2	0	14	0–8	< 0.001^a^ (vs saline placebo) 0.015^a^ (vs levobupivacaine)
	48	4	0	18	2–10	< 0.001^a^ (vs saline placebo) 0.021^a^ (vs levobupivacaine)
Saline placebo	12	10	4	38	6–16	ns
	24	16	6	50	10–22	0.003^a^ (vs ketorolac tromethamine) < 0.001^a^ (vs combination)
	48	24	8	76	16–30	0.001^a^ (vs ketorolac tromethamine) < 0.001^a^ (vs combination)

*CI* confidence interval

^a^significant difference among groups calculated using ANOVA/ Kruskal Wallis tests, and between groups comparison *post-hoc* tests with Bonferroni correction; ns, non-significant

There were significant differences in the total consumption of piritramide at 24 h post-caesarian section between the groups of KT versus placebo (*p* = 0.003), LB + KT versus placebo (*p* < 0.001) and LB versus LB + KT (*p* = 0.015). However, there were no significant differences in the total consumption of piritramide at 24 h between the other groups, as LB versus placebo (*p* = 1.00), KT versus LB + KT (*p* = 1.000) and LB versus KT (*p* = 0.141). Similarly, there were significant differences in the total consumption of piritramide at 48 h between the groups of KT versus placebo (*p* = 0.001), LB + KT versus placebo (*p* < 0.001) and LB versus LB + KT (*p* = 0.021). There were no significant differences in the total consumption of piritramide at 48 h between the other groups, as LB versus placebo (*p* = 1.000), KT versus LB + KT (*p* = 1.000) and LB versus KT (*p* = 0.054).

**Fig. 3:**
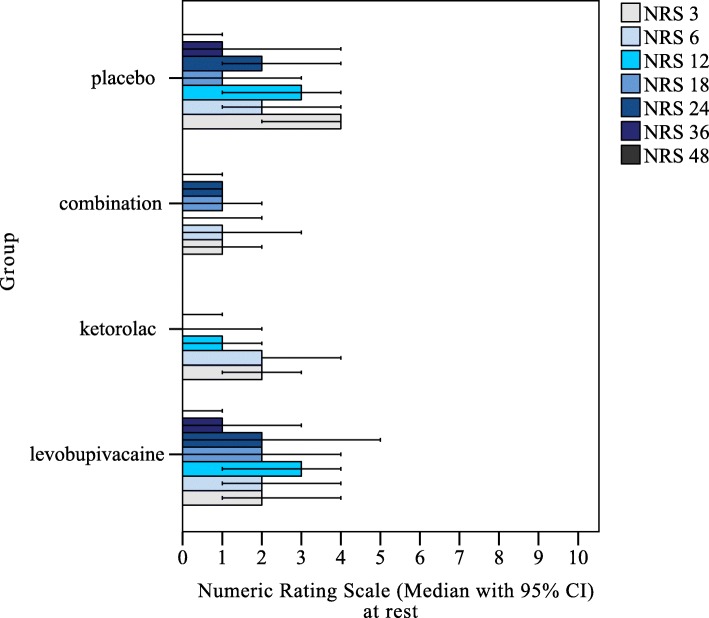
Medians and interquartile ranges across the four parturient groups for the NRS for pain at rest, at 3, 6, 12, 18, 24, 36 and 48 h post-caesarean section. *,° outliers

**Fig. 4:**
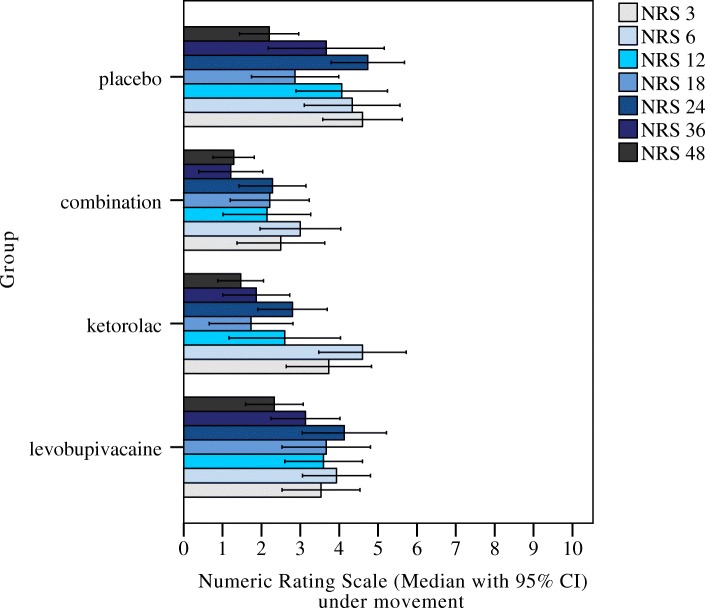
Medians and interquartile ranges across the four parturient groups for the NRS for pain under movement, at 3, 6, 12, 18, 24, 36 and 48 h post-caesarean section. *,° outliers

The data collected for the pain NRS for the seven time points (i.e., 3, 6, 12, 18, 24, 36, 48 h) at rest and under movement are shown in Figs. 3 and 4, respectively. There were significant differences for pain NRS at rest between the groups: after 3 h as LB + KT versus placebo (*p* = 0.006); after 12 h as LB + KT versus placebo (*p* = 0.015), and LB versus LB + KT (*p* = 0.046); after 24 h as KT versus placebo (*p* = 0.005), LB + KT versus placebo (*p* = 0.041), and LB versus KT (*p* = 0.035); and after 36 h as KT versus placebo (*p* = 0.013). There were no significant differences for pain NRS at rest for any of the other comparisons (Fig. 3). There were also significant differences for pain NRS under movement between the groups: after 24 h as KT versus placebo (*p* = 0.025), and LB + KT versus placebo (*p* = 0.003); after 36 h as LB + KT versus placebo (*p* = 0.018), and LB versus LB + KT (*p* = 0.029) (Fig. 4).

The recorded times from the end of the caesarean section to the first request for piritramide, for persistent post-operative pain (after 8 weeks), for any new skin sensations at the operative site (after 8 weeks), and for parturient satisfaction with the quality of their analgesia over the first 48 h post-caesarean section (after 72 h), are presented in Table 4. Persistent pain was reported by one parturient (7.1%) in the LB group and one parturient (7.1%) in the KT group. No parturients complained about persistent pain in the LB + KT group or in the placebo group. The feeling of new sensation was significantly different among the groups (*p* = 0.026). The most common new sensation was for the placebo group (60%), and the least common for the LB + KT group (7.1%). The quality of analgesia over 48 h post-operative time was assessed 72 h post-caesarean section. Parturients in the LB + KT group defined their analgesia as excellent, and in the placebo group as very good (*p* = 0.005).

**Table 4:** Pain-associated data across the four parturient groups

Parturient group	Time to first piritramide request [min (95% CI)]	Persistent post-operative pain (8 weeks; yes/no) [n (%)]	New skin sensation (8 weeks; yes/no) [n (%)]	Quality of analgesia (48 h; NRS 1–5)
Levobupivacaine	163 (120–250)	1 (7.1)	7 (46.7)	4 (4–5)
Ketorolac tromethamine	180 (160–465)	1 (7.1)	5 (35.7)	4 (4–5)
Combination	295 (125–475)	0 (0)	**1 (7.1)** ^**a**^	**5 (5–5)** ^**a**^
Saline placebo	160 (120–185)	0 (0)	**9 (60.0)** ^**a**^	**4 (4–5)** ^**a**^
*p*-value	**ns**	**	**0.026**^**a**^ (combination vs. saline placebo)	**0.005**^**a**^ (combination vs. saline placebo)

*CI* confidence interval

^a^significant difference among groups calculated using ANOVA/ Kruskal Wallis tests, and between groups comparison *post-hoc* tests with Bonferroni correction; ^**^p value could not be calculated due to zero positive cases in some groups; ns, non-significant; NRS 1–5, numeric rating scale for quality of analgesia from 1 to 5; significant differences are marked with bold text

## Discussion

This study compared the effectiveness of pain treatments with in-wound infusion of levobupivacaine and/or ketorolac and placebo after caesarean section. These data show more effective post-caesarean analgesia when ketorolac or the combination of levobupivacaine with ketorolac were used for the in-wound infusion. Furthermore, these data show that two parturients from the KT group and six from the LB + KT group did not need any piritramide in the first 24 h post-caesarean section, while one parturient from the KT group and five from the LB + KT group did not need any piritramide by 48 h post-caesarean section.

Local anaesthetics have an important role in post-operative analgesia, and they might have a role in the prevention of persistent pain after caesarean section [13, 14]. Some previous trials have provided evidence of analgesia benefits from adding NSAIDs to local anaesthetics for continuous in-wound infusion. Carvalho et al. showed that when low-dose ketorolac, but not hydromorphone, was added to 48-h continuous bupivacaine in-wound infusion, this significantly improved analgesia after caesarean section. Ketorolac reduced pain scores and the need for analgesia, and also reduced the inflammatory cytokines in the wound exudate (i.e., interleukines 6 and 10) [15]. Lavand’homme et al. investigated continuous in-wound infusion of diclofenac for post-caesarean section analgesia, as compared to ropivacaine and placebo, and combined with systemic diclofenac therapy. They reported that local infusion of diclofenac significantly reduced post-operative morphine consumption in comparison with saline infusion and systemic diclofenac, without any specific adverse effects. Thus, post-operative analgesia produced by local diclofenac infusion was as effective as local ropivacaine infusion with systemic diclofenac [16].

Rackelboom et al. evaluated the significance of the anatomical layer (i.e., above the fascia, below the fascia) for continuous in-wound infusion of ropivacaine combined with ketoprofene, using a multiorifice catheter. They showed that infusion below the fascia significantly reduced both pain at rest and total post-operative morphine consumption, compared with infusion above the fascia [17].

However, some studies have reported no specific advantages of continuous infusion of local anaesthetics below the fascia [18], while others have reported that subfascial continuous in-wound infusion of local anaesthetics for post-operative analgesia is as effective as intrathecal morphine [19, 20], or even more effective than epidural morphine [21]. O’Neil et al. reported that their ropivacaine continuous in-wound infusion group achieved better analgesia than their epidural morphine group, with significantly lower side effects and less staff workload [21]. Kainu et al. reported that continuous in-wound infusion with ropivacaine or saline for 48 h was less effective than intrathecal morphine, with no difference between the local anaesthetic and placebo groups [18]. Their findings support our results, with no significant difference in analgesic efficacy between levobupivacaine and placebo.

Adesope et al. provided a systematic review and meta-analysis of 21 studies on local anaesthetics for in-wound infiltration for post-caesarean analgesia. They reported reduced pain scores at rest and under movement and significant reduction in opioid consumption at 24 h with catheter placement below the fascia, as opposed to above the fascia [22]. Our data are not in agreement here comparing the anatomical placement of the catheter, where we used an NSAID in combination with the local anaesthetic.

There are some concerns about continuous subcutaneous infusion of ketorolac. There have been no studies on ketorolac pharmacokinetics after subcutaneous infusion in pregnant or postpartum women. This will probably be slightly different from healthy volunteers. However, there is some evidence of this use in cancer patients and healthy volunteers, and it appears to be safe [23, 24]. In the present study, the maximal allowed daily doses for i.v. therapy for post-caesarean section analgesia were used, on the assumption that subcutaneous infusion would be as safe as i.v. therapy. Subcutaneous infusion has been reported to result in lower high-peak plasma levels in healthy volunteers, and as such, this administration of ketorolac might have fewer side-effects [24].

There are a number of limitations to this study. The primary outcome of the study was total amount of rescue piritramide, as sufficient to achieve optimal post-caesarean section analgesia. Achieving NRS ≤4 might not have been good enough for all of these parturients, and there was no upper limit to the rescue piritramide dose at any time. Functional recovery is maybe the most important issue after caesarean section.

During the study period, the Department policy for obstetric spinal anaesthesia was changed from 0.5% levobupivacaine to 0.5% hyperbaric bupivacaine. The study protocol was not changed, but the spinal anaesthesia block was seen to be less successful with levobupivacaine in this study (i.e., anaesthesia not deep enough, or block not great enough for surgery). Indeed, nine (15.3%) of the parturients needed supplemental analgesics and/or general anaesthesia, as most of them reached a block to only TH_6_, and one for the extended operation. All of these parturients were included in the analysis, as this situation did not activate any of the specified exclusion criteria.

However, the parturient numbers that were reached here were not as many as initially defined in the sample size calculation, as the study was terminated early for ethical reasons (i.e., not to expose our parturients to any unnecessary pain or/and general anaesthesia). Thus, an important limitation here is that our study was terminated early, with smaller numbers of parturients included. Indeed, this might have been the causal factor for the significant differences in the parturient characteristics concerning age and gestational age, between the LB group and the placebo group. Therefore this study can be considered as a pilot study.

Also at this stage, the ideal effective dose of ketorolac for addition to local anaesthetics for in-wound infusion is not known. Further studies are needed to evaluate the full clinical advantages of ketorolac added to a local anaesthetic for in-wound infusion for post-caesarean section analgesia.

To the best of our knowledge, the present study is the first trial to investigate the efficacy of over-fascia continuous in-wound infusion of a local anaesthetics and a NSAID separately and in combination, in comparison to saline placebo. Based on these data, ketorolac alone and in combination with a local anaesthetic as in-wound infusion are the most effective for reduction of post-operative consumption of piritramide. The novelty of our study lies in our data for successful post-caesarean section analgesia with a combination of a local anaesthetic and a NSAID for over-the-fascia in-wound infusion.

## Conclusion

The present study indicates that local infiltration of a NSAID alone or in combination with a local anaesthetic have superior efficacy over a local anaesthetic alone for reduction of opioid consumption during post-caesarean section analgesia.

## Abbreviations

ASA: American Society of Anesthesiologists
CI: confidence interval
CONSORT: Consolidated Standards of Reporting Trials
i.v.: intravenously
KT: ketorolac tromethamine
LB: levobupivacaine
NRS: numeric rating scale
NSAID: non-steroidal anti-inflammatory drug
SD: standard deviation
SpO_2_: peripheral oxygen saturation pressure
